# N/P-Dependent DNA Complexation, Transfection, and Cytotoxicity of Imine-Linked Low-Molecular-Weight PEI Polyplexes

**DOI:** 10.3390/ijms27167444

**Published:** 2026-08-20

**Authors:** Vera-Maria Platon, Vlad Ghizdovat, Iolanda Augustin, Ramona Lungu, Constantin Volovat, Diana-Ioana Panaite, Madalina Raluca Ostafe, Cristian Constantin Volovat, Dragos-Ioan Rusu, Lacramioara Ochiuz, Maricel Agop, Andiana Roxana Blidari, Simona Ruxandra Volovat

**Affiliations:** 1 Laboratory of Polycondensation and Thermostable Polymers, Petru Poni Institute of Macromolecular Chemistry of Romanian Academy, 700487 Iasi, Romania; platon.vera@icmpp.ro (V.-M.P.); lungu.ramona@icmpp.ro (R.L.); 2 Department of Biophysics and Medical Physics, Faculty of Medicine, Grigore T. Popa University of Medicine and Pharmacy, 16 University Street, 700115 Iasi, Romania; 3 Department of Pathophysiology and Immunology, Faculty of Medicine, Carol Davila University of Medicine and Pharmacy Bucharest, 050474 Bucharest, Romania; 4 Department of Medical Oncology-Radiotherapy, Faculty of Medicine, Grigore T. Popa University of Medicine and Pharmacy, 16 University Street, 700115 Iasi, Romania; cvolovat@gmail.com (C.V.);; 5 Department of Radiology, Faculty of Medicine, Grigore T. Popa University of Medicine and Pharmacy, 16 University Street, 700115 Iasi, Romania; 6 Department of Environmental Engineering, Faculty of Engineering, “Vasile Alecsandri” University of Bacau, 600115 Bacau, Romania; drusu@ub.ro (D.-I.R.);; 7 Department of Pharmaceutical Sciences, Faculty of Pharmacy, Grigore T. Popa University of Medicine and Pharmacy, 16 University Street, 700115 Iasi, Romania; 8 Academy of Romanian Scientists, Ilfov, 050044 Bucharest, Romania; 9 Faculty of Medicine, “Victor Babeș” University of Medicine and Pharmacy, 300041 Timișoara, Romania

**Keywords:** non-viral vectors, imine, polyplex, DNA transfection, nanomedicine, cancer

## Abstract

Gene delivery with cationic polymers requires balancing DNA compaction, colloidal stability, and intracellular release, yet for imine-linked low-molecular-weight polyethyleneimine (PEI) vectors, quantitative relationships connecting the N/P ratio with the full property–transfection cascade remain undefined. Here, two amphiphilic non-viral vectors were prepared by linking a hydrophobic benzene–siloxane core (TAS) to hyperbranched PEI (800 or 2000 Da) through reversible imine bonds and complexed with DNA across a broad N/P range (10–600). Polyplexes were characterized by atomic force microscopy (AFM), dynamic light scattering (DLS), ζ-potential, agarose gel electrophoresis, transfection via green fluorescent protein (GFP) imaging and luciferase assay in HeLa cells. Both vectors formed spherical nano-entities (AFM diameters ~30 nm for TAS-PEI800; ~100 nm for TAS-PEI2000). TAS-PEI2000 achieved complete DNA retardation at N/P ≈ 30 versus N/P ≈ 150 for TAS-PEI800, consistent with its higher charge density (ζ = +37.59 vs. +18.35 mV). Transfection efficiency was superior for TAS-PEI2000 across most N/P ratios; however, TAS-PEI2000 displayed an optimal transfection efficiency at N/P ≈ 100 (ζ ≈ 3.84 mV), beyond which efficiency declined, indicating a binding–release trade-off. Cell viability remained >77% across the N/P range for TAS-PEI800, but dropped below 25% at N/P ≥ 400 for TAS-PEI2000. A phenomenological logistic model identified characteristic transition thresholds (θ ≈ 60 for TAS-PEI800; θ ≈ 40 for TAS-PEI2000), capturing the onset of cooperative self-assembly; however, the post-optimum decline observed for TAS-PEI2000 requires additional inhibitory terms. These findings demonstrate that PEI molecular weight governs both the N/P threshold required for efficient transfection and the width of the therapeutic window, thereby providing structure–activity descriptors for the rational design of imine-linked polyplex systems.

## 1. Introduction

Gene therapy relies on delivering exogenous genetic material into target cells to correct or modulate the expression of defective genes. Given the breadth of diseases linked to genetic alterations, from cancer and cardiovascular disease to neurodegenerative conditions, the development of effective delivery vehicles remains a central challenge [1,2]. These vehicles, or vectors, are broadly classified as viral (modified viruses) or non-viral (synthetic compounds), each carrying distinct advantages and trade-offs [3,4].

Non-viral vectors are attractive because of their low pathogenicity, capacity for chemical modification, and scalable production. Their principal limitation, however, is the low transfection efficiency [5]. A wide range of non-viral platforms have been explored in recent years, including polymer-based systems; dendrimer-based carriers, which offer precise structural control and multivalency; lipid-based systems such as lipid nanoparticles and liposomes, which have demonstrated clinical success in nucleic acid delivery; and inorganic nanoparticles, which provide tunable physicochemical properties and stability [6,7,8,9]. Despite these advances, polymer-based systems remain highly attractive due to their synthetic versatility and ease of functional modification.

Among non-viral platforms, polymer-based systems, particularly polyplexes formed between cationic polymers and DNA, have received considerable attention [10]. PEI remains the most widely studied cationic polymer for gene delivery, largely owing to its strong DNA condensation and proton-sponge-mediated endosomal escape. However, PEI’s clinical translation has been consistently limited by a molecular-weight-dependent toxicity–efficiency trade-off: high-MW variants (25 kDa) transfect effectively but cause dose-dependent cytotoxicity through membrane disruption and mitochondrial damage, while low-MW variants (≤2 kDa) are safer but inefficient as standalone carriers [11,12].

Three principal strategies have been pursued to resolve this blockage. First, crosslinking low-MW PEI through biodegradable linkages, including disulfide bonds [13], ester linkages [14], or cleavable conjugation to inorganic or polymeric scaffolds [15,16,17,18], yields architectures that combine the condensation capacity of larger networks with the safety profile of short chains. Second, chemical modification with amino acids such as histidine, tyrosine, and lysine [19,20], cell-penetrating peptides [21], or PEGylated supramolecular platforms [22] has been reported to improve cellular uptake and biocompatibility in various cell models. Third, dynamic covalent chemistry, specifically reversible imine bonds linking low-MW PEI to hydrophilic PEG blocks [23,24,25] or to hydrophobic Jeffamine and polysiloxane cores [26,27], has been shown to enable pH-responsive assembly and disassembly, providing extracellular stability with triggered intracellular release. This last approach is particularly compelling because it simultaneously addresses both the toxicity and the cargo-release barrier, yet a quantitative understanding of how imine-linked architecture governs the relationship between N/P ratio, colloidal properties, and transfection remains limited.

Computational and mathematical modeling approaches have increasingly been applied to guide non-viral vector design, ranging from molecular dynamics and quantitative structure–activity relationship (QSAR) to machine learning-based prediction of cell-penetration efficiency [28,29,30]. However, these efforts have focused primarily on lipid nanoparticles or on predicting individual physicochemical endpoints in isolation. For imine-linked PEI systems, no study has provided a unified quantitative framework connecting the N/P ratio across the full cascade, from colloidal properties to biological output. In this work, we report the synthesis of two amphiphilic non-viral vectors (TAS-PEI800 and TAS-PEI2000) linked through reversible imine bonds, characterize their polyplexes over a broad N/P range (10–600), and introduce a phenomenological nonlinear model that identifies transition thresholds governing self-assembly and transfection efficiency.

## 2. Results

### 2.1. Synthesis of Non-Viral Vectors and Polyplex Formation

**Figure 1:**
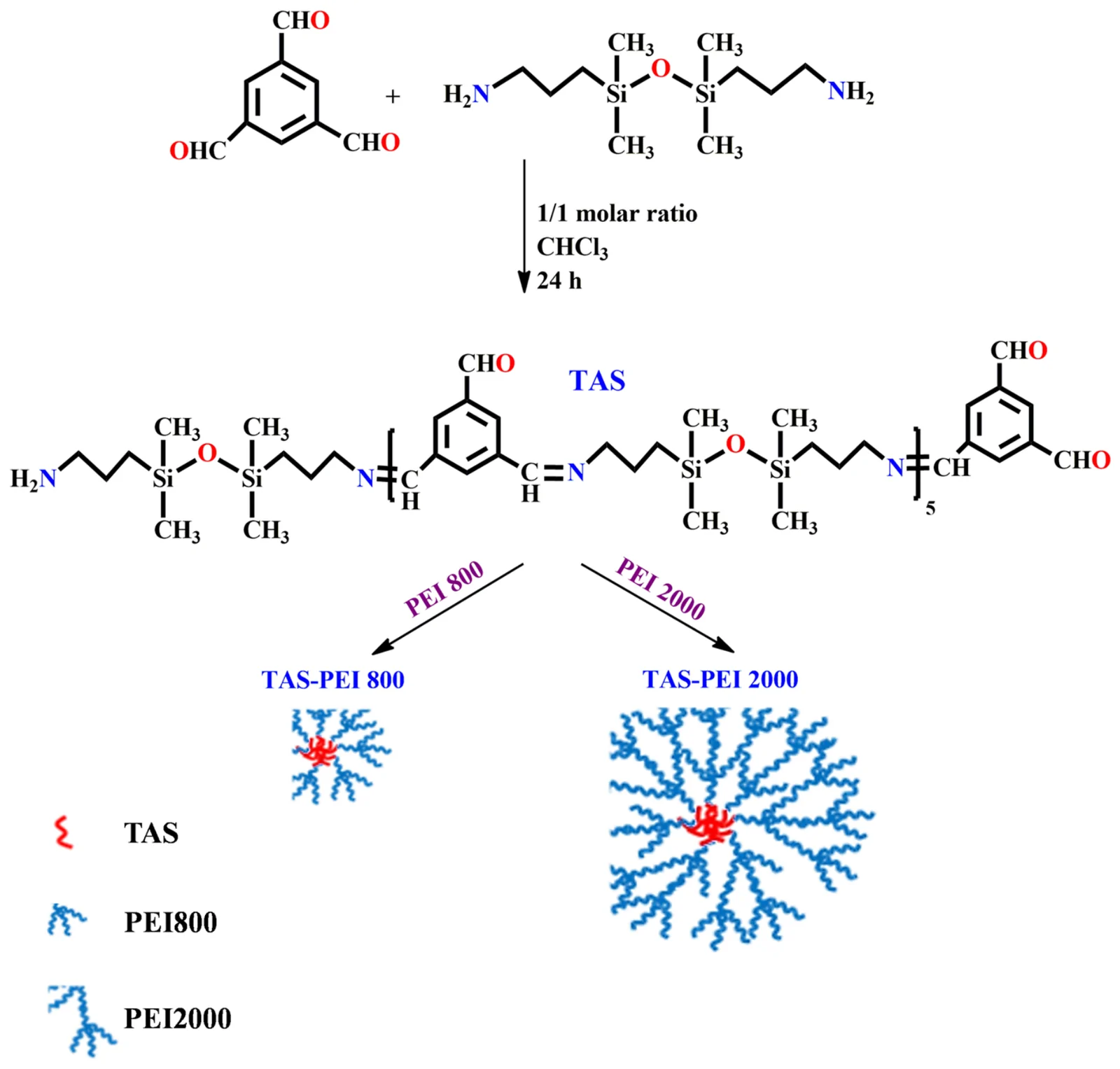
Synthetic pathway for TAS-PEI800 and TAS-PEI2000.

**Figure 2:**
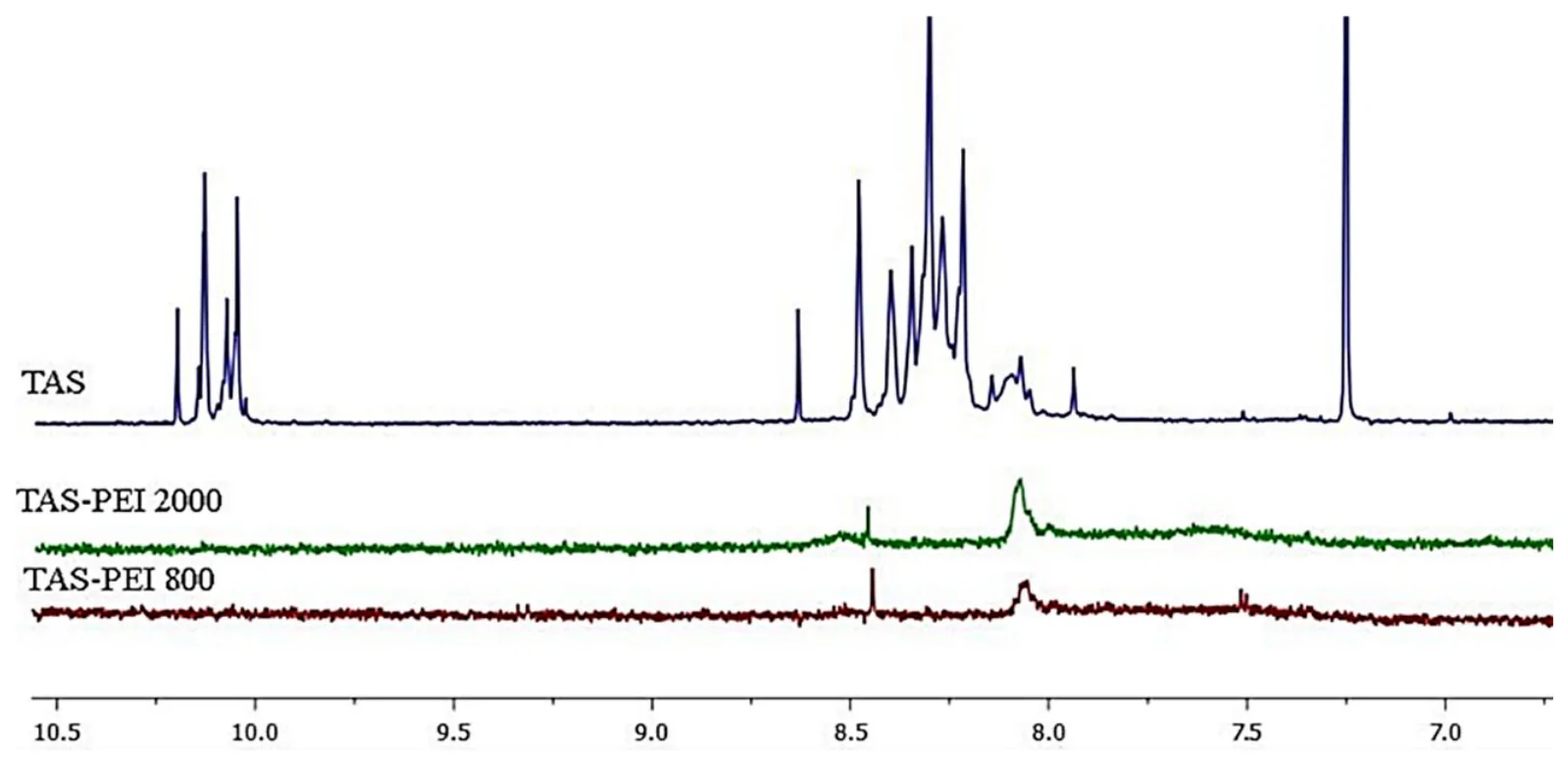
Fragments of ^1^H NMR spectra of TAS (CDCl_3_, 400 MHz, 25 °C), TAS-PEI800, and TAS-PEI2000 compounds (D_2_O, 400 MHz, 25 °C).

The synthetic route to TAS-PEI800 and TAS-PEI2000 is outlined in Figure 1. The successful synthesis of TAS-PEI800 and TAS-PEI2000 was confirmed by nuclear magnetic resonance (NMR) and fourier-transform infrared (FTIR) spectroscopy. The ^1^H NMR spectrum of TAS revealed two signals at 10.15 and 10.1 ppm corresponding to monosubstituted and disubstituted aldehyde protons, while signals in the aliphatic region were assigned to the siloxane unit. In the 8–9 ppm region, multiple bands corresponding to aromatic ring protons and newly formed imine linkages were observed (Figure 2, Figure S1) [31].

The NMR spectra of TAS-PEI800 and TAS-PEI2000 showed complete disappearance of the aldehyde signals. Imine protons appeared as a sharp band at 8.4 ppm and a broad band around 8.5 ppm, while aromatic protons were observed at 8.0–8.2 ppm (Figure 2, Figure S1).

Upon gradual acidification, a signal corresponding to aldehyde protons appeared at approximately ~10.0 ppm, indicating the reversibility of the imine linkage under acidic conditions (Figure S2) [32]. No significant signs of degradation were observed over a pH range of 1.0–9.0 (Figure S3). The minor chemical shift changes can be attributed to the protonation of functional groups and associated changes in the electronic environment.

**Figure 3:**
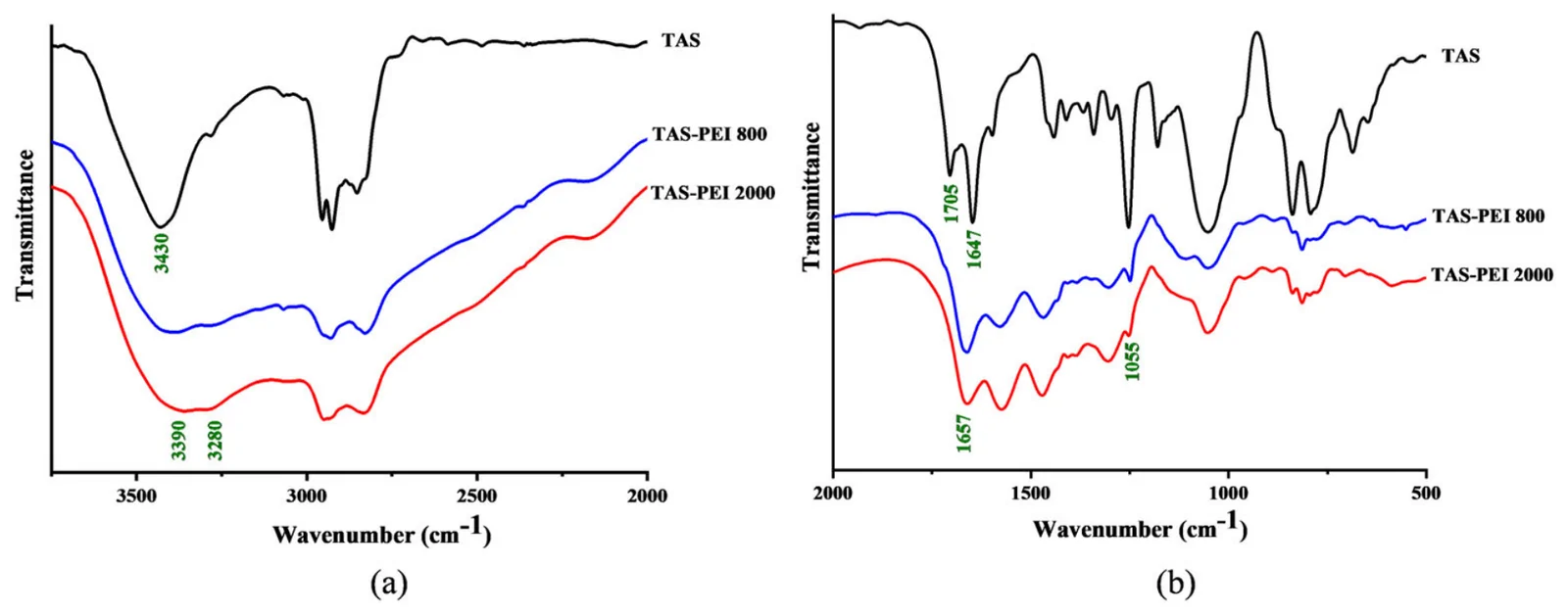
FTIR spectra of the studied compounds in (**a**) 3500–2000 and (**b**) 2000–500 spectral domains.

FTIR spectra displayed a strong absorption at 1647 cm^−1^ (TAS) and 1657 cm^−1^ (TAS-PEI800 and TAS-PEI2000), assigned to C = N stretching (Figure 3). The TAS spectrum also revealed a band at 1705 cm^−1^ corresponding to free aldehyde groups, which disappeared after condensation with PEI.

Polyplexes were generated by combining TAS-PEI800 or TAS-PEI2000 with DNA at N/P ratios from 10 to 600 (Table 1). Salmon sperm DNA was used for physicochemical characterization, while pEGFP plasmid DNA was used for transfection experiments, as in previous work [24]. Agarose gel electrophoresis showed progressive decrease in electrophoretic mobility with increasing N/P ratio (Figure S4). Complete DNA retardation was achieved at N/P = 150 for TAS-PEI800 (P5) and N/P = 30 for TAS-PEI2000 (P2*).

### 2.2. Morphological Characterization

**Figure 4:**
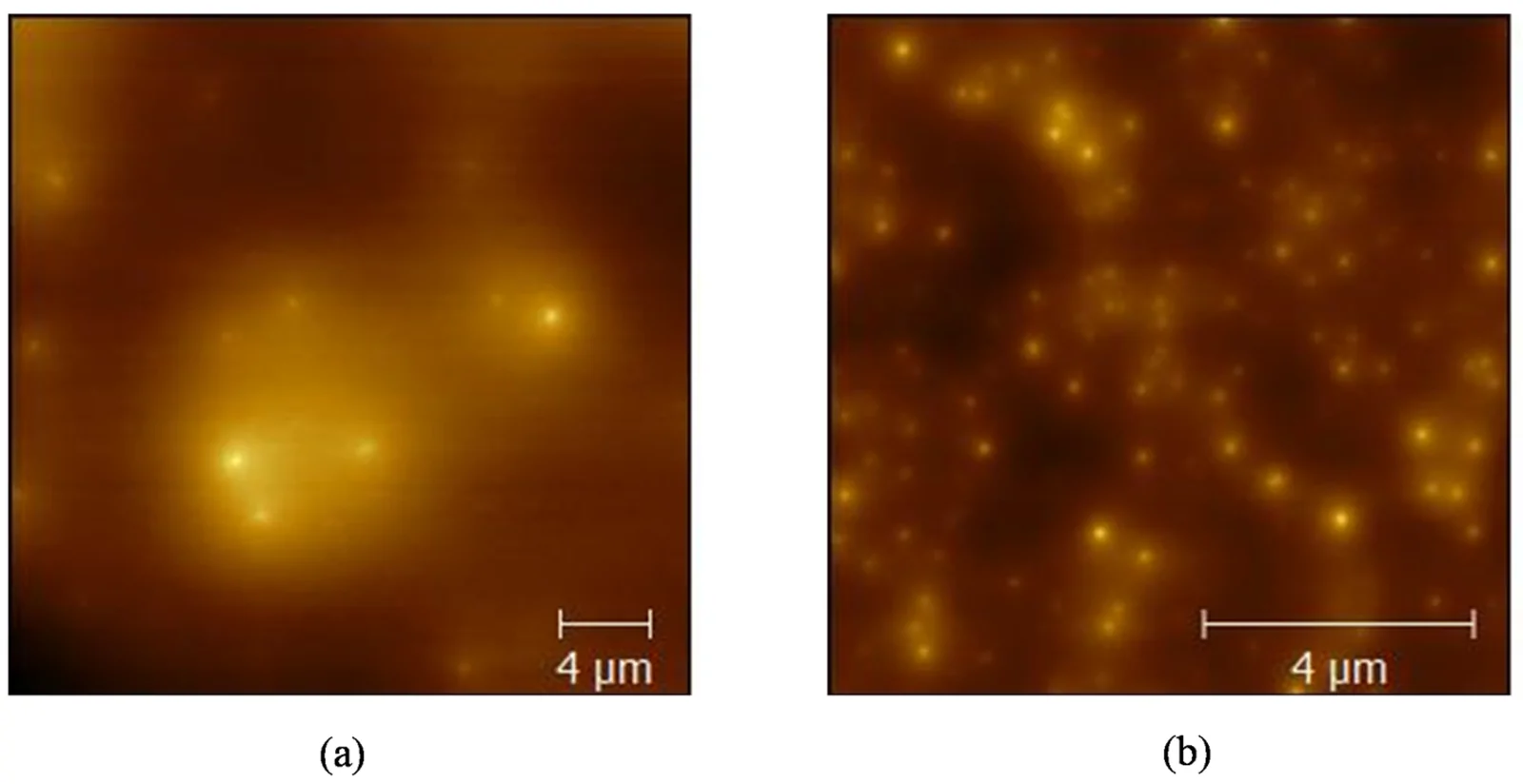
AFM images of (**a**) TAS-PEI800 and (**b**) TAS-PEI2000.

AFM images revealed that both TAS-PEI800 and TAS-PEI2000 formed spherical nano-entities in aqueous solution. TAS-PEI800 generated smaller structures with a mean diameter of ~30 nm, while TAS-PEI2000 formed larger nano-entities with a mean diameter of ~100 nm (Figure 4).

**Figure 5:**
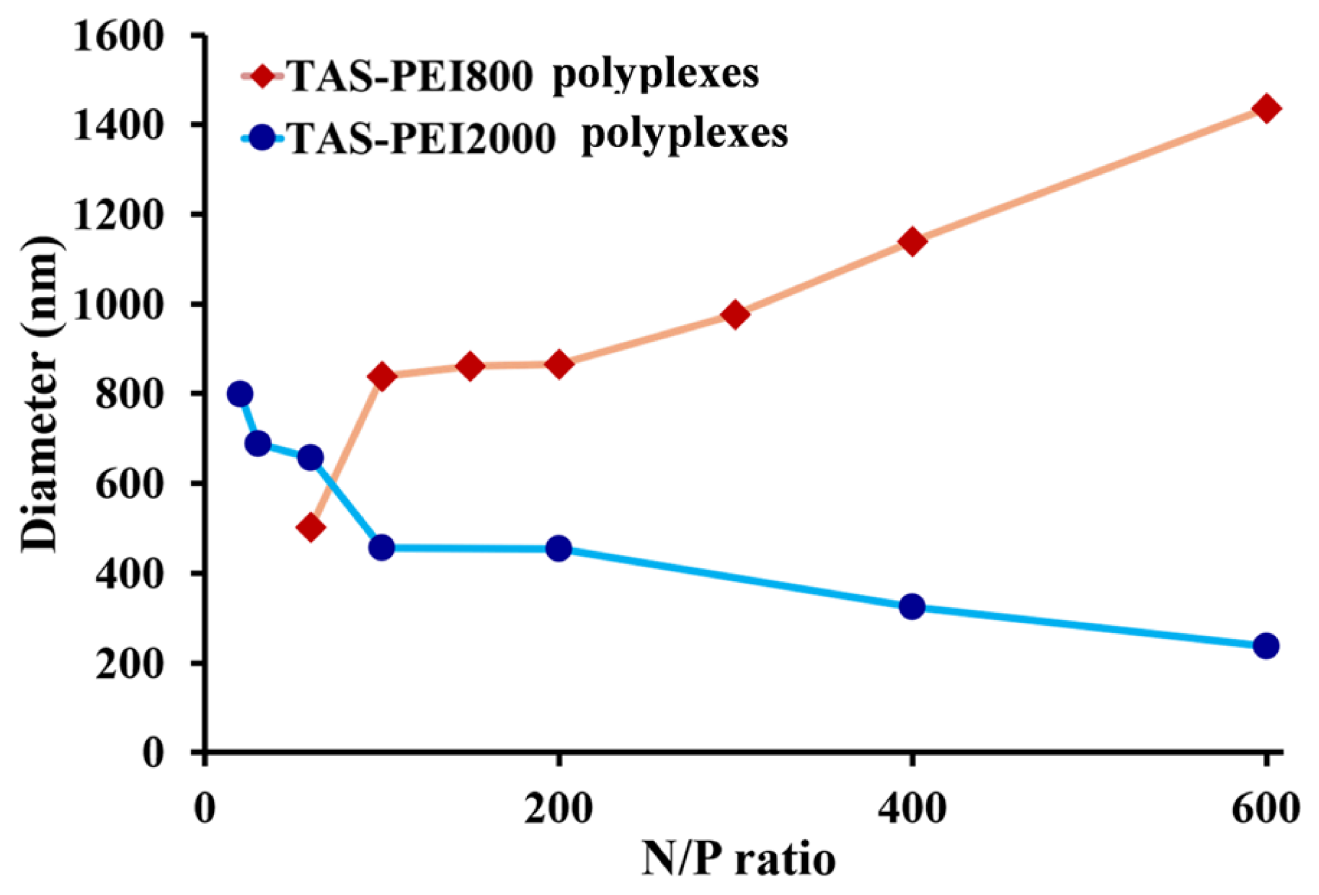
DLS measurements of TAS-PEI800 and TAS-PEI2000 polyplexes.

DLS measurements confirmed the formation of the two nanoentities by the two amphiphils, with hydrodynamic diameters of 487.8 nm and 871.4 nm for TAS-PEI800 and TAS-PEI2000, respectively. The hydrodynamic diameters were revealed to be larger than AFM sizes for both systems (Figure 5). The two vector series showed divergent trends upon DNA complexation: for TAS-PEI800, polyplex diameter progressively increased with increasing N/P ratio, exceeding the size of the free vector. For TAS-PEI2000, the opposite was observed: polyplex size decreased with increasing N/P ratio, producing particles smaller than the free vector.

**Figure 6:**
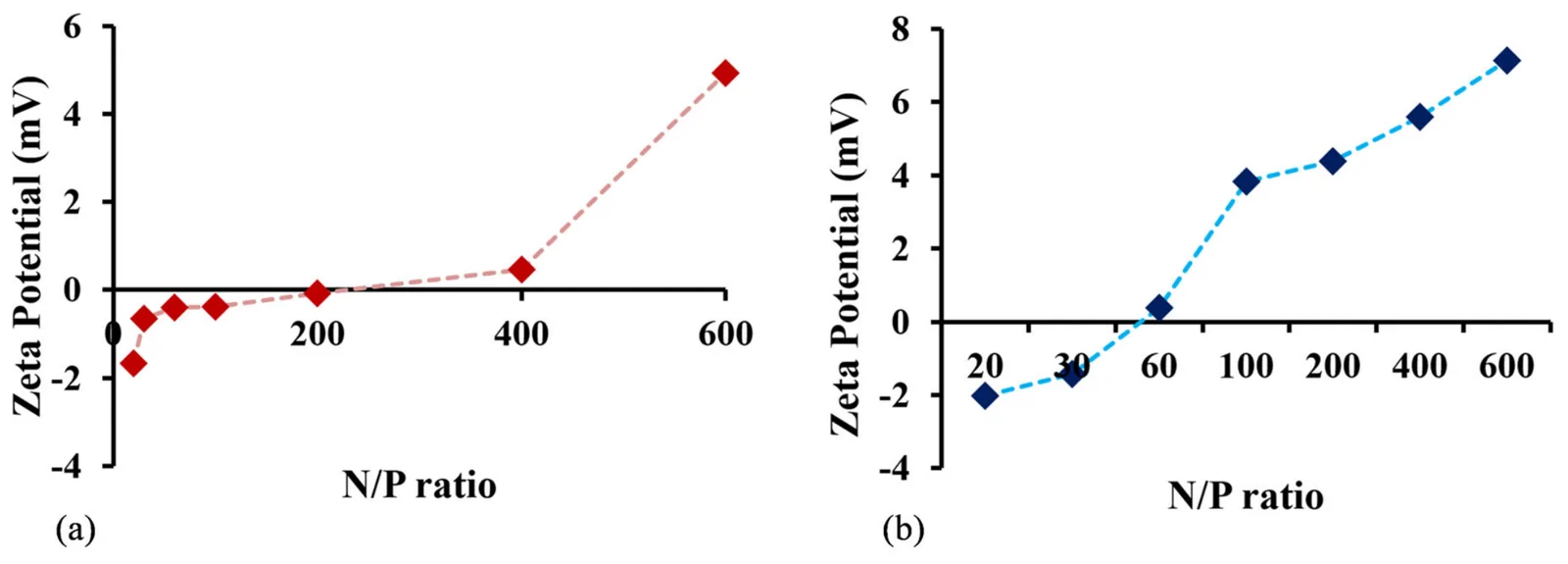
Zeta Potential values of (**a**) TAS-PEI800 and (**b**) TAS-PEI2000 polyplexes.

ζ-potential values for the free vectors were +18.35 mV (TAS-PEI800) and +37.59 mV (TAS-PEI2000). Upon DNA complexation, ζ-potential decreased. At lower N/P ratios, polyplexes were anionic, indicating an excess of uncompensated DNA charge. With increasing N/P, ζ-potential shifted through neutrality and into the cationic regime for both systems (Figure 6).

### 2.3. Transfection Efficiency of the Polyplexes

**Figure 7:**
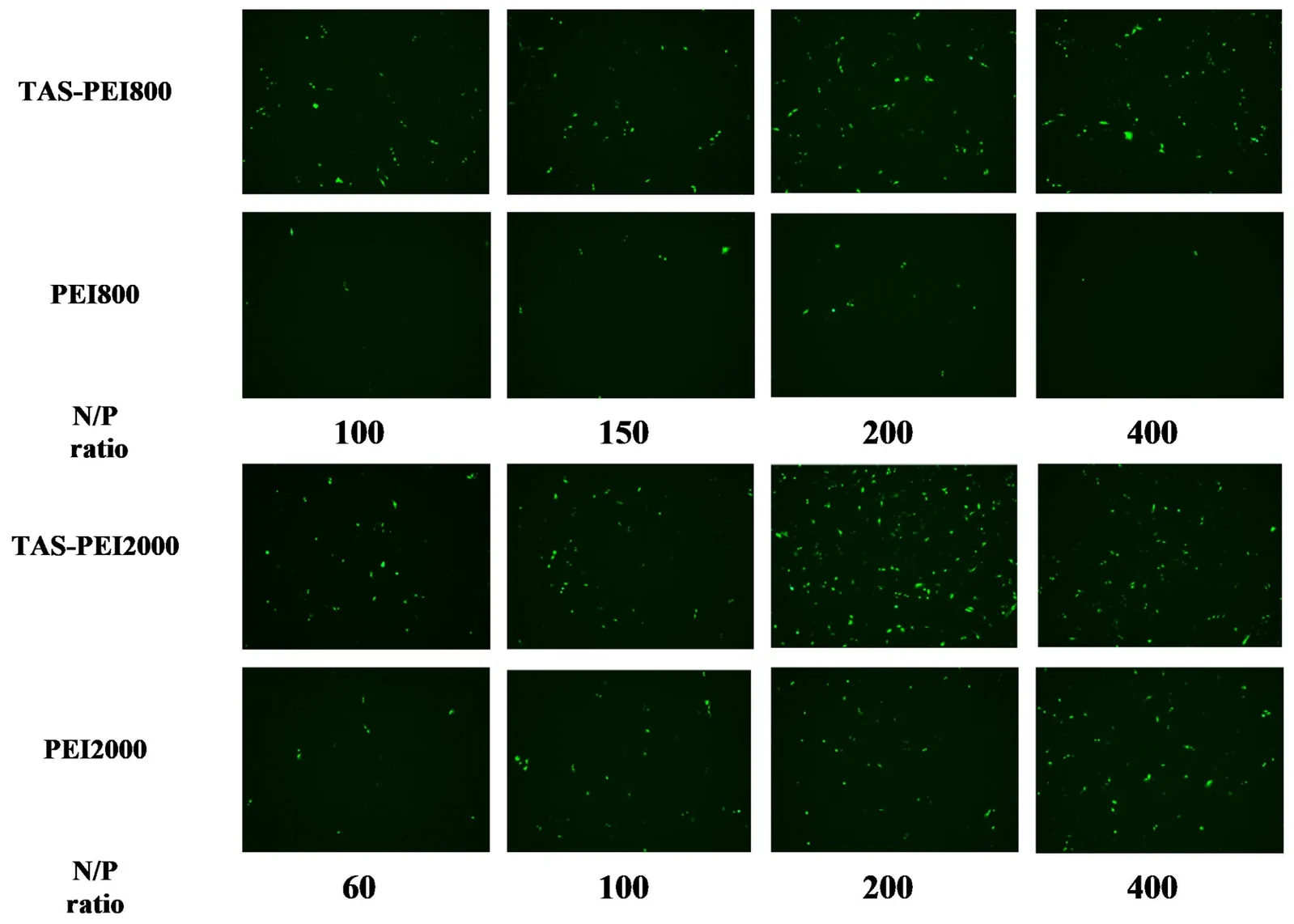
The visualization of the green fluorescent protein in HeLa cells.

Transfection was first assessed qualitatively by fluorescence microscopy. GFP expression increased with N/P ratio for both vector systems and was visibly higher than for unmodified PEI 800 Da and PEI 2000 Da controls across all tested ratios (Figure 7). TAS-PEI2000 polyplexes produced stronger GFP signals than TAS-PEI800 at equivalent N/P ratios.

**Figure 8:**
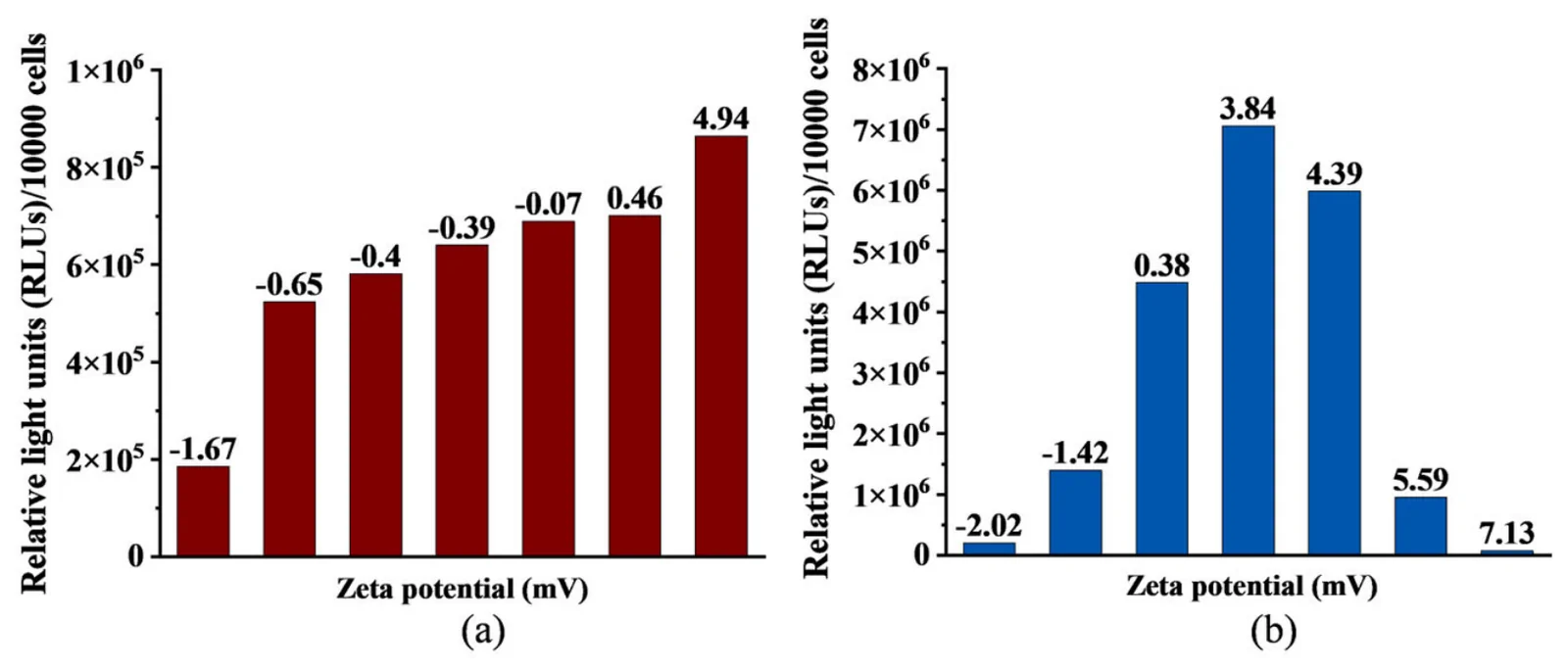
The transfection efficiency on HeLa cells for TAS-PEI800 (**a**) and TAS-PEI2000 (**b**) polyplexes.

Quantitative luciferase assays confirmed these observations (Figure 8). For TAS-PEI800, transfection efficiency increased progressively with ζ-potential across the tested N/P range. For TAS-PEI2000, efficiency increased with ζ-potential up to 3.84 mV (corresponding to N/P = 100), beyond which a decline was observed.

Cell viability data (Table 2) showed that TAS-PEI800 polyplexes maintained viability above 77% across the entire N/P range tested. TAS-PEI2000 polyplexes showed a progressive viability decrease at higher N/P ratios, dropping to 81.3% at N/P = 100 and below 25% at N/P ≥ 400. This trend indicates a clear dependence of cellular response on the N/P ratio, with higher ratios leading to reduced cell viability. These findings suggest the existence of an optimal N/P range in which sufficient cellular integrity is preserved to enable effective transfection while maintaining measurable biological activity.

## 3. Discussion

This study demonstrates that PEI molecular weight governs both the cooperative self-assembly threshold and the width of the effective transfection window in imine-linked amphiphilic polyplex systems. TAS-PEI2000 achieved complete DNA retardation at substantially lower N/P (30 vs. 150), exhibited higher transfection efficiency, and showed a more abrupt cooperative transition (θ ≈ 40 vs. 60) than TAS-PEI800. However, TAS-PEI2000 also displayed a more narrow therapeutic window, with viability dropping below 25% at N/P ≥ 400 (Table 2), whereas TAS-PEI800 maintained viability above 77% across the entire range tested. The non-monotonic transfection profile observed for TAS-PEI2000, with a distinct optimum at N/P ≈ 100 (ζ ≈ 3.84 mV), suggests a binding–release trade-off that limits efficiency beyond a critical charge threshold.

These findings are consistent with the broader PEI literature, where molecular weight is recognized as the principal determinant of the efficiency–toxicity balance [11,12,33]. The divergent self-assembly trajectories observed here, with TAS-PEI800 polyplexes expanding and TAS-PEI2000 polyplexes compacting with increasing N/P, mirror the general observation that higher charge density promotes tighter DNA condensation at the cost of reduced intracellular release [12]. The discrepancy between AFM and DLS diameters (~30 vs. ~100 nm by AFM; larger by DLS) is attributable to the different measurement conditions: AFM captures dried particle dimensions while DLS reflects the hydrodynamic diameter of solvated, swollen PEI chains in aqueous solution [34].

Compared with the cyclic siloxane-PEI vectors reported by Uritu et al. [27], which employed 25 kDa PEI and achieved optimal transfection at N/P 10–40, the present systems require substantially higher N/P ratios, reflecting the reduced charge density of low-MW PEI (800 and 2000 Da). Conversely, the dynamic imine architecture confers a potential release advantage absent from permanently crosslinked systems: acid-catalyzed imine cleavage provides a built-in disassembly trigger in the endosomal environment (pH 5.0–5.5), consistent with the pH-dependent structural reorganization recently documented for PEI polyplexes by Uddin et al. [35]. The imine-linked systems previously reported by our group using PEG blocks [23,24] or Jeffamine cores [26] demonstrated improved transfection relative to unmodified PEI, but did not provide quantitative descriptors linking N/P to the property–transfection cascade. The logistic threshold parameters (θ, k) introduced here represent a first attempt at such a unified description.

Beyond the comparison with siloxane-based architectures, the present systems differ from the broader family of PEI-based vectors in the mechanism by which assembly and release are decoupled. Unmodified PEI25k remains the efficiency benchmark but carries dose-dependent membrane and mitochondrial toxicity, whereas low-MW PEI (≤2 kDa) is well tolerated yet inefficient as a standalone carrier [11,12]. Strategies that restore condensation capacity by crosslinking low-MW PEI through disulfide or ester bridges [13,14] or by conjugation to inorganic scaffolds [15] depend on a reducing or esterase-rich intracellular environment for cargo release, while PEGylation improves colloidal stability at the cost of charge shielding and reduced cellular uptake [22]. The imine linkage used here couples both functions to a single bond: it drives amphiphilic self-assembly at neutral pH and is cleaved in the endosomal pH range (5.0–5.5), providing an intrinsic release trigger without requiring a reductive or enzymatic stimulus [23,24,25,35]. Two further practical advantages follow: transfection was retained in serum-containing medium, a condition that typically suppresses PEI-mediated delivery, and the choice of PEI block (800 vs. 2000 Da) tunes the width of the therapeutic window, a design handle unavailable in single-architecture systems. The trade-off is the higher N/P ratio required relative to high-MW PEI vectors.

The logistic model adequately captured the cooperative onset of transfection for both systems, but its limitations must be acknowledged. The low MSE for TAS-PEI800 (0.47) confirms a good fit across the tested N/P range, whereas the high MSE for TAS-PEI2000 (74.99) demonstrates that a monotonic logistic function cannot describe the post-optimum decline (Table 3). This decline most likely reflects the combined effect of over-compaction, which hinders endosomal disassembly and DNA release, and direct cytotoxicity at high cationic charge density, as evidenced by the viability data (Table 2). A more complete model would require either an additional inhibitory term or a piecewise formulation separating the cooperative assembly regime from the toxicity-dominated regime.

The non-monotonic E(ζ) relationship, with an optimum near ζ ≈ 3–5 mV, further supports the interpretation that moderate surface charge optimizes the balance between cellular uptake and intracellular release, while excessive charge becomes counterproductive.

Notably, all transfection experiments were performed in serum-containing medium (alpha-MEM with 10% FBS), indicating that TAS-PEI polyplexes maintain functional activity under conditions that typically inhibit PEI-mediated gene delivery through protein corona formation and charge neutralization [12,33]. This represents a practical advantage for potential translational applications.

However, several limitations should be acknowledged. First, physicochemical characterization was performed with salmon sperm DNA while transfection used plasmid DNA; size and ζ-potential may differ between these substrates due to differences in molecular weight and topology [36]. Second, transfection was evaluated in a single cell line (HeLa); the optimal N/P window is likely cell-type-dependent, as uptake mechanisms and endosomal processing vary between cell types [12]. Third, no in vivo data are available; the high N/P ratios required (60–100) may raise toxicity concerns in systemic administration, though the viability data suggest a manageable therapeutic window for TAS- PEI800 across the tested range. Fourth, the pH-dependent imine cleavage and endosomal release mechanism, while thermodynamically plausible and consistent with the vector design, was not experimentally validated in this study.

Despite these limitations, the present work provides quantitative structure–activity descriptors (θ, k, D_max) that could inform rational optimization of imine-linked polyplex formulations. The identification of system-specific N/P thresholds and optimal ζ-windows moves beyond the empirical trial-and-error approach that dominates current polyplex design, toward a framework where colloidal and transfection properties can be predicted from vector architecture.

## 4. Materials and Methods

### 4.1. Materials

Benzene-1,3,5-tricarboxaldehyde (Manchester Organics, Runcorn, Cheshire, UK), 1,3-bis-(3-aminopropyl)–1,1,3,3–tetramethyldisiloxane, and hyperbranched polyethylenimine (MW = 800 Da and 2000 Da, 50 wt% in H_2_O) were purchased from Sigma-Aldrich Chemie (Taufkirchen, Germany). Salmon sperm DNA was obtained from Fluka (Buchs, Switzerland). All reagents were used as received.

Cell culture plates were from Corning (Corning, NY, USA). Alpha-MEM, penicillin-streptomycinamphotericin B mixture, and Trypsin-Versene were from Lonza (Basel, Switzerland); fetal bovine serum (FBS) from Gibco Thermo Fisher Scientific (Grand Island, NY, USA); HeLa cells from CLS Cell Lines Service GmbH (Eppelheim, Germany). CellTiter 96 AQueous One Solution Cell Proliferation Assay (MTS) kit and Bright-Glo Luciferase Assay System kit were aquired from Promega (Madison, WI, USA).

Plasmids pCS2+MT-Luc (pLuc), encoding firefly luciferase, and pCS2+NLS-eGFP (pEGFP), encoding enhanced green fluorescent protein, were a gift from Prof. Adrian Salic (Harvard University, MA, USA). Plasmids were amplified in *E. coli* DH5α (gift from Dr. Anca Gafencu, “Nicolae Simionescu” Institute of Cellular Biology and Pathology, Bucharest, Romania) and purified using the E.Z.N.A. Endo-Free Plasmid Mini II kit (Omega Bio-Tek, Inc., Norcross, GA, USA).

### 4.2. Synthesis of Non-Viral Vectors

The non-viral vectors were synthesized following a previously reported procedure [24]. Briefly, a hydrophobic core was first prepared by reacting benzene-1,3,5-tricarboxaldehyde with 1,3-bis-(3-aminopropyl)-1,1,3,3-tetramethyldisiloxane at a 1:1 molar ratio in chloroform. The resulting compound was designated TAS. In the second step, TAS was reacted with hyperbranched polyethylenimine of two molecular weights (800 and 2000 Da), yielding TAS-PEI800 and TAS-PEI2000, respectively. These reactions were carried out in a THF/ethanol mixture at a final concentration of 1%. The synthetic pathway is shown in Figure 1.

### 4.3. Formation of Polyplexes

Polyplexes were prepared by mixing TAS-PEI800 or TAS-PEI2000 with DNA at N/P molar ratios ranging from 10 to 600. Salmon sperm DNA was used for physicochemical characterization (AFM, DLS, ζ-potential, gel electrophoresis), while plasmid DNA (pEGFP/ pLuc) was used for transfection experiments. The N/P ratio was defined as the molar ratio of protonatable nitrogen atoms in the vector to phosphate groups in the DNA backbone, assuming 3 nmol of phosphorus per μg of DNA. The nitrogen content was calculated from the PEI fraction in each vector and determined to be 19.7% for TAS-PEI800 and 26.8% for TAS-PEI2000. Polyplexes prepared with TAS-PEI800 were labeled P1–P6, and those prepared with TAS-PEI2000 were labeled P1*–P6* (Table 1).

### 4.4. Equipment

NMR spectroscopy was performed on a Bruker Avance DRX 400 MHz spectrometer (Bruker Corporation, Billerica, MA, USA) equipped with a 5 mm QNP probe with z-gradients, at room temperature. The TAS sample was prepared in CDCl_3_, while TAS-PEI 2000 and TAS-PEI 800 were prepared in D_2_O. For the imine reversibility studies, 37% aqueous HCl was gradually added to the TAS-PEI 2000 and TAS-PEI 800 samples, in order to adjust the pH within the range of 1.0–9.0. The upper limit corresponds to the initial pH values measured for the respective components in D_2_O. Chemical shifts are reported as δ values (ppm) relative to the residual solvent peak.

FTIR spectra were recorded on KBr pellets using a Bruker Vertex 70 spectrophotometer (Bruker Optik GmbH, Ettlingen, Germany) over the 4000–600 cm^−1^ range, with 32 scans and 4 cm^−1^ resolution.

Atomic force microscopy (AFM) was performed using an Ntegra Spectra instrument (NT-MDT, Moscow, Russia) in semicontact mode at room temperature with an NSG 10 silicon cantilever. Samples were prepared by depositing 5 μL of suspension onto freshly cleaved mica, allowed to dry, and rinsed with deionized water to remove residual salts.

Gel retardation assay. Polyplex formation was assessed by agarose gel electrophoresis. DNA and polyplex samples were mixed with loading buffer and loaded onto a 1% agarose gel. Electrophoresis was carried out at 90 V for 120 min in TAE buffer. Gels were stained with ethidium bromide and visualized under UV illumination.

Zeta potential and DLS measurements were performed at 25 °C using a Delsa Nano C Submicron Analyzer (Beckman Coulter, Brea, CA, USA) in deionized water. DLS and ζ-potential measurements were performed in triplicate, each replicate representing the average of 70 instrumental runs as per the instrument software protocol. Transfection and cell viability assays were performed as three independent biological experiments.

Cell culture. HeLa cells were maintained as monolayers in alpha-MEM supplemented with 10% FBS and 1% penicillin-streptomycin-amphotericin B at 37 °C in a humidified atmosphere containing 5% CO_2_.

Fluorescence microscopy. Transfection was qualitatively assessed by fluorescence microscopy 48 h post-transfection using a Leica DMI 3000B (Leica Microsystems, Wetzlar, Germany) inverted microscope equipped with a GFP filter cube.

Transfection studies. HeLa cells were seeded in 96-well opaque microplates at 10^4^ cells/well in 100 μL complete medium. After 24 h, the medium was replaced with complete medium (alpha-MEM supplemented with 10% FBS) containing polyplexes formed with pLuc plasmid at a final DNA concentration of 5 ng/μL. After 48 h incubation, 100 μL/well Bright- Glo reagent (Promega, Madison, WI, USA) was added and luminescence was measured using an Ensight multimode plate reader (PerkinElmer, Waltham, MA, USA).

Cell viability. Viability was evaluated under identical conditions on HeLa cells using the MTS assay (CellTiter 96 AQueous One Solution, Promega). Relative viability was calculated as a percentage of untreated control cells (Table 2).

The software used for data acquisition and analysis included MestReNova (version LITE, Mestrelab Research S.L., Santiago de Compostela, Spain), TopSpin 4.0 (Bruker, Billerica, MA, USA), and OriginPro 2021 (OriginLab Corporation, Northampton, MA, USA).

### 4.5. Mathematical Modeling

In non-viral delivery systems, the physicochemical properties of the vector can no longer be described within a linear framework, since each particle responds differently to variations in charge density, pH, and molecular conformation. In the case of TAS-PEI vectors, self-assembly in the presence of DNA leads to a sequence of successive stability states, which is a hallmark of nonlinear systems. Small variations in the N/P ratio may therefore induce abrupt changes in particle size, zeta potential, or transfection efficiency (Table 4).

The vector-DNA complexation process can be regarded as an evolution toward a minimum free energy state:

(1) ΔG=ΔH−TΔS<0,

where ΔG is the free energy, ΔH is the enthalpy, T is the absolute temperature and ΔS is the entropy.

The entropic contribution is significant: binding of DNA restricts the degrees of freedom of the polymer chains, but this conformational penalty can be energetically compensated by the formation of electrostatic interactions.

The dominant electrostatic contribution can be approximated by:

(2) $$E_{\mathrm{coul}}=\frac{1}{4\pi \varepsilon }\frac{q_{PEI}\cdot q_{DNA}}{r},$$

where $E_{\mathrm{coul}}$ is a Coulombic interaction energy between vector and DNA charges, ε is the effective dielectric permittivity of the medium, $q_{PEI}$ is the effective net positive charge of PEI, $q_{DNA}$ is the effective net negative charge of DNA, and r is the charge-charge separation distance.

Thus, it can be observed that even small variations in the interaction distance or in the effective charge may lead to macroscopically observable effects, such as the neutralization of electrophoretic mobility.

Experimental data indicate that particle size does not increase linearly with the N/P ratio. Instead, the evolution follows a logistic-type behavior:

(3) $$D\left(\frac{N}{P}\right)=\frac{D_{min}}{1+e^{-k\left[\left(\frac{N}{P}\right)-\theta \right]}}$$

(4) $$D\left(\frac{N}{P}\right)=\frac{D_{max}}{1+e^{-k\left[\left(\frac{N}{P}\right)-\theta \right]}},$$

where D is the particle size, k is a logistic steepness parameter and θ is the logistic threshold. For TAS-PEI800, the growth process is slow (small k), and particles expand gradually. For TAS-PEI2000, the organization is rapid, and the system undergoes a sudden transition toward a compact structure.

Such logistic behavior is typical for self-assembled structures, in which crossing a certain charge threshold triggers a complete reorganization of the polymeric surface.

The zeta potential of the nanoparticles plays a crucial role in both colloidal stability and interaction with the cellular membrane. Its evolution can be approximated by:

(5) $$\zeta \left(\frac{N}{P}\right)=\zeta_{0}+\alpha \left(\frac{N}{P}\right)-\beta I,$$

where $\zeta_{0}$ is the intrinsic surface potential, α is the charge-density coefficient, β is the ionic-screening coefficient, and I is the ionic strength.

Equations (3)–(5) provide a general theoretical framework for the expected behavior; fitted parameters are reported only for the transfection efficiency model.

Experimental curves reveal the existence of a critical point: beyond a certain N/P ratio, further increases in positive charge no longer enhance transfection, but instead reduce it. This behavior is characteristic of nonlinear systems exhibiting a local optimum.

Imine bonds undergo cleavage in the acidic endosomal environment:

(6) $$\frac{d\left[C_{imine}\right]}{dt}=-k_{acid}\left[C_{imine}\right],$$

(7) $$C_{DNA}\left(t\right)=C_{0}\left(1-e^{-k_{\mathrm{acid}}t}\right),$$

where $C_{imine}$ is the imine bond concentration, $k_{\mathrm{acid}}$ is the first-order rate constant for acid-catalyzed cleavage, $C_{DNA}\left(t\right)$ is the released DNA concentration at time t, and $C_{0}$ is the initial complexed DNA concentration.

As the pH decreases, a sudden structural reorganization occurs: the nanoparticle transitions from a compact state to a dispersed configuration, releasing DNA. This abrupt change is typical of systems governed by an instability threshold.

These equations describe the expected release kinetics; experimental validation of pH-dependent disassembly was not performed in the present study and remains a target for future work.

A comparison of dynamic differences between TAS-PEI2000 and TAS-PEI800 can be found in Table 4.

**Figure 9:**
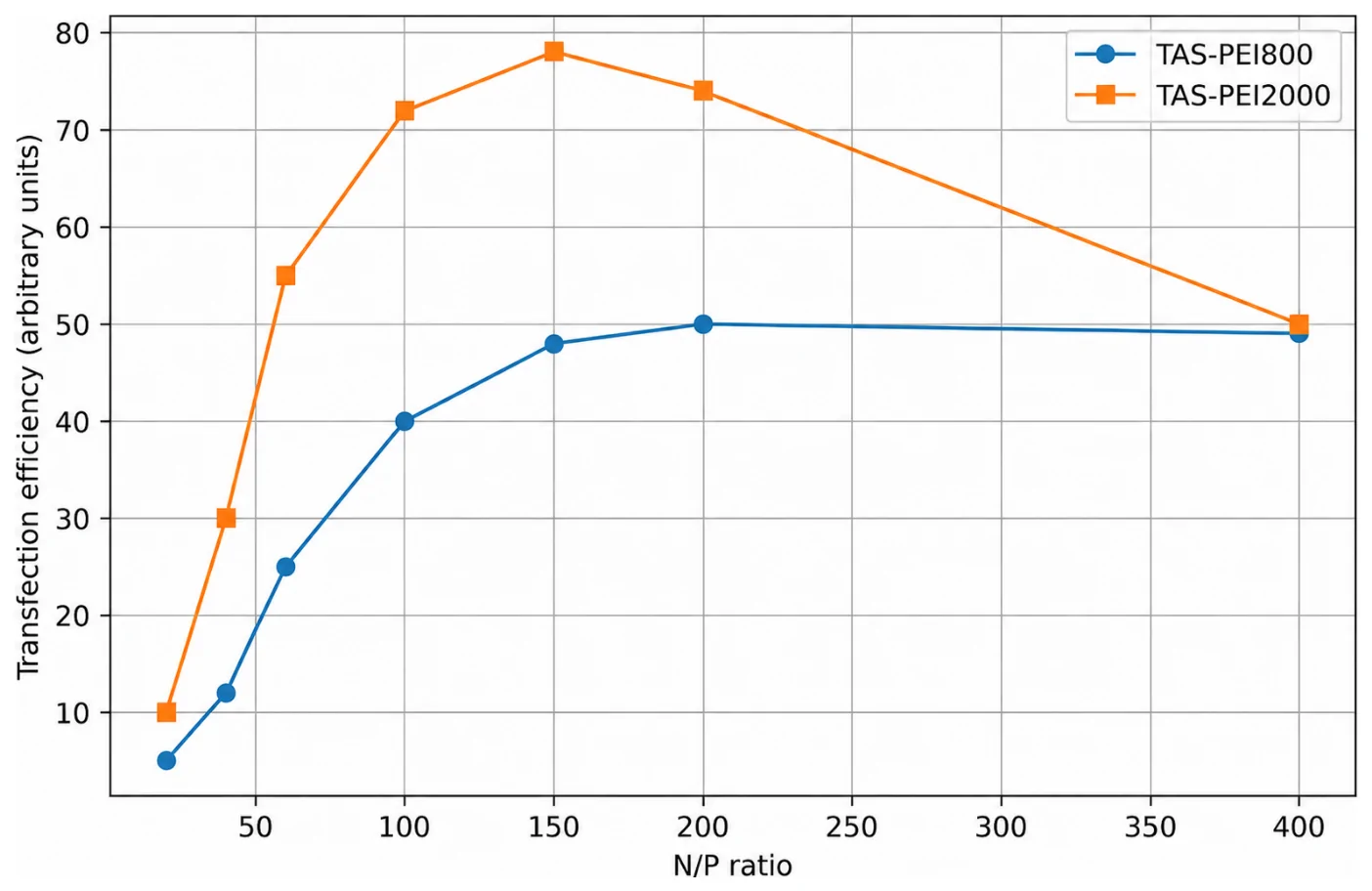
Nonlinear behavior of transfection efficiency.

TAS-PEI2000 behaves as a dense network with a high density of protonable “nodes”. As a result, DNA complexation induces a rapid collapse and the formation of an optimal polyplex already at low N/P ratios. The experimentally dependence of transfection efficiency on N/P ratio is shown in Figure 9. Furthermore, the logistic-model parameters for transfection efficiency can be found in Table 3.

The transfection efficiency was modeled as a function of the N/P ratio using a logistic model:

(8) $$E\left(\frac{N}{P}\right)=\frac{D_{max}}{1+\exp\left[-k\left(\frac{N}{P}-\theta \right)\right]}.$$

**Figure 10:**
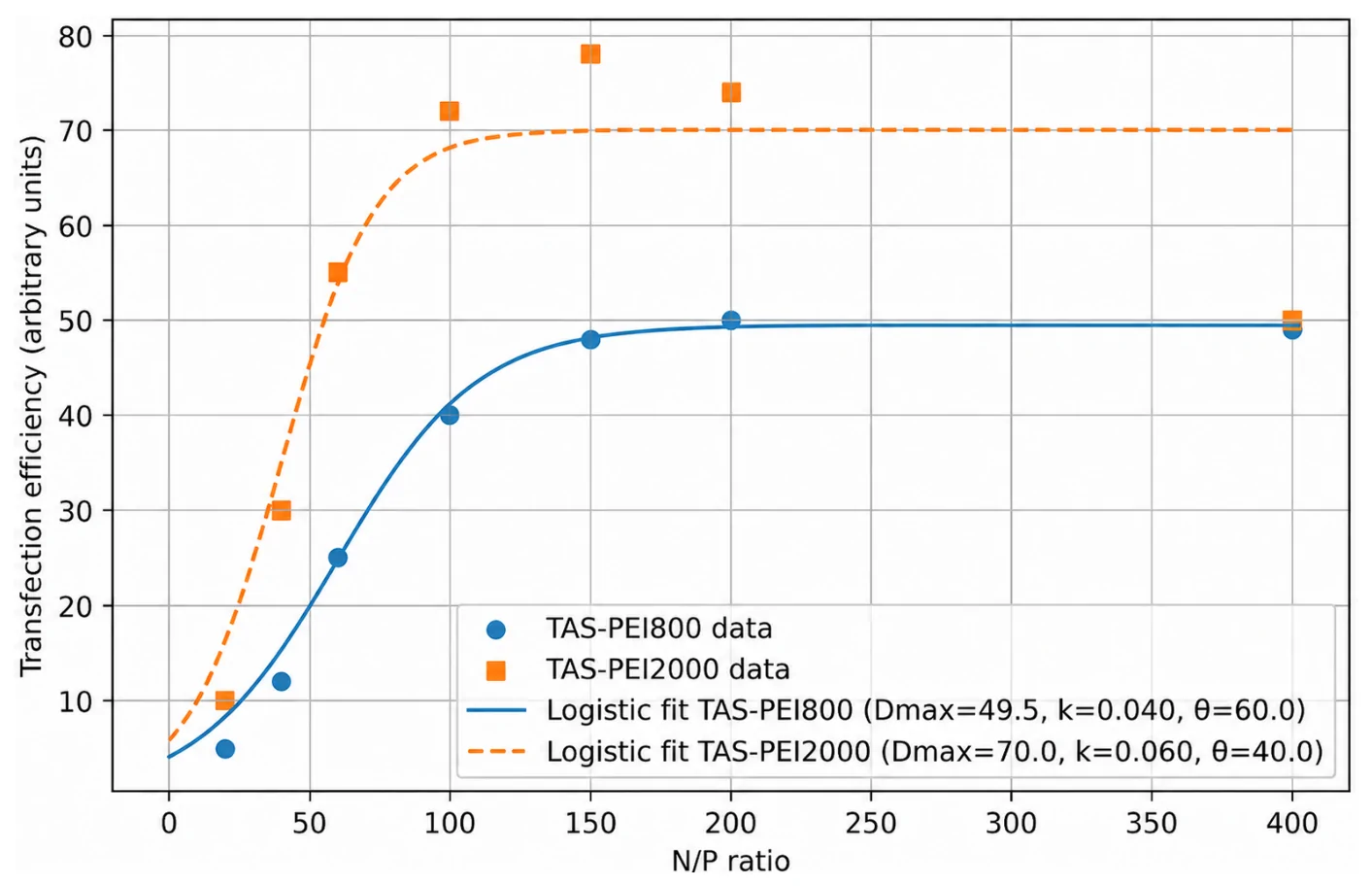
Logistic fit of transfection efficiency.

The corresponding logistic fit is presented in Figure 10.

Grid-search fitting yielded the following parameter estimates (approximate values, arbitrary units) for TAS-PEI800:

(9) $$D_{\max\;}\approx 49.5,k\approx 0.040,\theta \approx 60$$

The system exhibits a slower increase (small k) and reaches a moderate plateau, which is consistent with the observation that TAS-PEI800 requires higher N/P ratios to achieve efficient charge neutralization and compaction. TAS-PEI2000:

(10) $$D_{max}\approx 70.0,k\approx 0.060,\theta \approx 40$$

The denser network of protonable groups leads to an earlier transition (smaller θ) and to a higher plateau (larger $D_{max}$). This reflects a rapid cooperative organization and a superior efficiency prior to saturation.

The curves clearly demonstrate the nonlinear character of the process: small variations in the N/P ratio around θ produce pronounced jumps in transfection efficiency.

**Figure 11:**
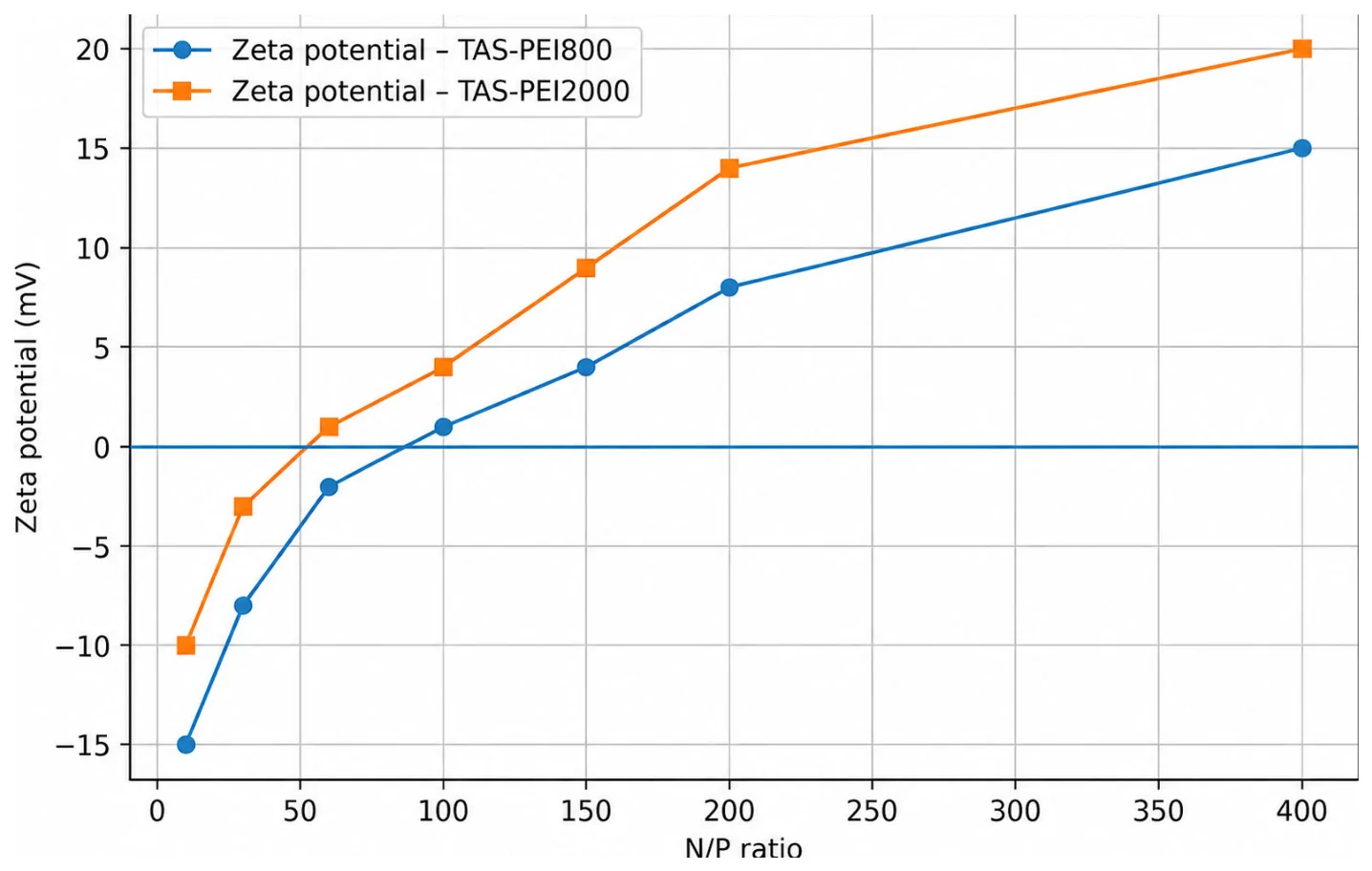
Evolution of the Zeta potential as a function of the N/P ratio.

As it can be seen from Figure 11, the zeta potential increases from negative values (anionic polyplex) toward zero and then into the positive domain, with an earlier crossover point for TAS-PEI2000 (≈60–100) and a later one for TAS-PEI800 (≈100–150). For TAS- PEI2000 the transfection efficiency increases together with the zeta potential up to approximately ~3.8 mV (N/P ≈ 100), after which it tends to decrease, suggesting the emergence of a trade-off between colloidal stability and intracellular release.

**Figure 12:**
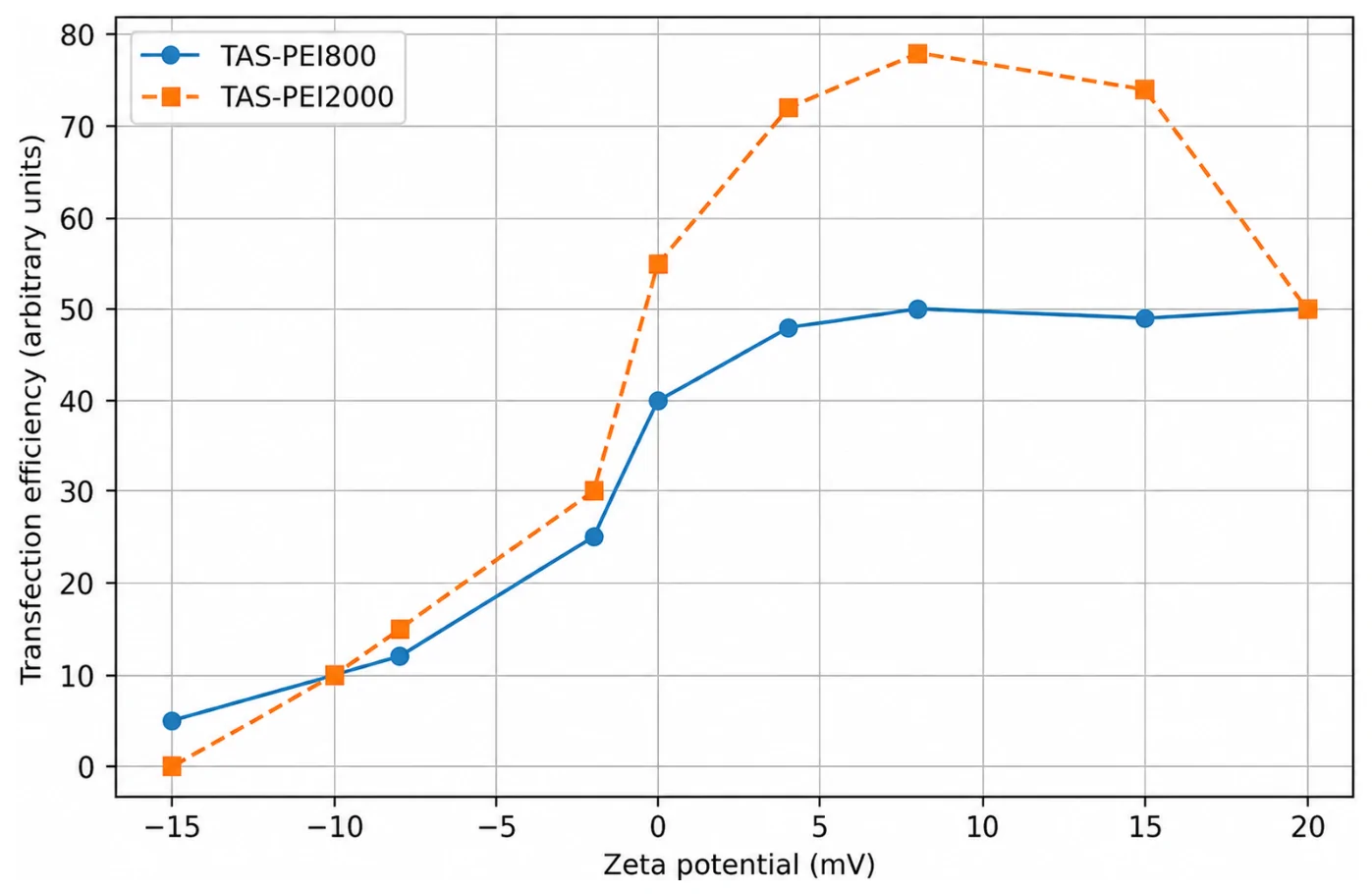
Transfection efficiency as a function of the zeta potential.

As shown in Figure 12, the *E*(*ζ*) curves are non-monotonic: an increase in positive surface charge initially enhances transfection (through improved adsorption, DNA protection, and endocytosis), but beyond a local optimum (approximately 3−8 mV) the efficiency levels off or decreases, most likely due to: (i) over-compaction (which hinders endosomal disassembly), (ii) excessively strong membrane interactions and/or local toxicity, (iii) more pronounced ionic screening, leading to reduced diffusion. This interpretation is consistent with the relative cell viability data shown in Table 2, which reveal a progressive loss of viability at higher N/P ratios, particularly for TAS-PEI2000.

This profile is typical of nonlinear systems governed by a critical threshold and by competition between energetic terms (electrostatic versus entropic contributions). For TAS-PEI2000, the high density of cationic groups reduces θ (earlier transition), while the larger slope k indicates more abrupt jumps between microstates; the local optimum coincides with the region ζ ≈ 3–5 mV, where the balance between DNA protection and endosomal release is most favorable. For TAS-PEI800, a larger θ and a smaller k indicate a more gradual organization process; the lower plateau suggests that the reduced charge density limits efficient compaction and translocation.

Taking the above into account, we can say that, rather than a monotonic increase with N/P, there exists an optimal window specific to each system, beyond which further charge addition becomes counterproductive (over-stabilization and hindered release).

## 5. Conclusions

Two amphiphilic non-viral vectors (TAS-PEI800 and TAS-PEI2000) were prepared via reversible imine linkages and formed DNA polyplexes across a broad N/P range (10–600). PEI molecular weight governed the entire property–transfection cascade: TAS-PEI2000 achieved complete DNA retardation at lower N/P, higher transfection efficiency, and a more abrupt cooperative transition (θ ≈ 40 vs. 60), but at the cost of a narrower therapeutic window, with viability falling below 25% at N/P ≥ 400 compared to >77% for TAS-PEI800 across the full range. The non-monotonic transfection profile of TAS-PEI2000, with a distinct optimum at N/P ≈ 100 (ζ ≈ 3.84 mV), demonstrates a binding–release trade-off that defines the upper boundary of useful charge density. A phenomenological logistic model captured the cooperative assembly onset for both systems, though the post-optimum decline for TAS- PEI2000 requires additional modeling terms. The quantitative descriptors identified here (θ, k, $D_{max}$, optimal ζ-window) provide a framework for rational optimization of imine-linked polyplex formulations, moving beyond empirical N/P screening toward predictive structure–activity relationships.

## Figures and Tables

**Table 1:** The N/P ratio for each polyplex and the corresponding code.

TAS-PEI2000	Polyplex	TAS-PEI800	Polyplex
N/P = 20	P1*	N/P = 10	P1
N/P = 30	P2*	N/P = 20	P2
N/P = 60	P3*	N/P = 50	P3
N/P = 100	P4*	N/P = 100	P4
N/P = 200	P5*	N/P = 150	P5
N/P = 400	P6*	N/P = 200	P6

**Table 2:** Relative Cell Viability Values Obtained for Different N/P Ratios.

TAS-PEI2000		TAS-PEI800	
Relative Cell Viability (%)	NP	Relative Cell Viability (%)	NP
100.75	20	105.3	60
99.7	30	103.4	100
97.3	60	101.2	150
81.3	100	90.5	200
58.7	200	89.2	300
24.3	400	92.3	400
18.3	600	77.4	600

**Table 3:** Logistic-model parameter estimates for transfection efficiency vs. N/P ratio for TAS-PEI800 and TAS-PEI2000 polyplexes ($D_{max}$, k, θ) and associated fitting error (MSE).

MSE	θ (N/P, Logistic Threshold)	k (1/N/P Unit)	$D_{max}$ (Units)	System	No.
0.47435	60	0.04	49.5	TAS-PEI800	**1**
74.99036	40	0.06	70	TAS-PEI2000	**2**

**Table 4:** Dynamic Differences Between TAS-PEI800 and TAS-PEI2000.

Property	TAS-PEI800	TAS-PEI2000
**Number of cationic groups**	Lower	Higher
**Particle size** (**AFM, vector nano-entities**)	~30 nm	~100 nm
**Stable polyplex formation threshold**	High N/P	Low N/P
**Reorganization type**	Slow, gradual	Abrupt, critical
**Maximum transfection**	Moderate	High

## Data Availability

The original contributions presented in this study are included in the article/Supplementary Material. Further inquiries can be directed to the corresponding author.

## Supplementary Materials

The following supporting information can be downloaded at: https://www.mdpi.com/article/10.3390/ijms27167444/s1.
