# Supplementary material for: N/P-Dependent DNA Complexation, Transfection, and Cytotoxicity of Imine-Linked Low-Molecular-Weight PEI Polyplexes
Source: Int J Mol Sci. 2026 Aug 20;27(16):7444. doi: 10.3390/ijms27167444 (PMC13513381; doi:10.3390/ijms27167444)
Supplement: Supplementary file 1 [file ijms-27-07444-s001.zip › ijms-4444946-supplementary.pdf]

Supporting material for

**Dynamic Imine-Linked Amphiphilic PEI Polyplexes for Non-Viral Gene Delivery: Morphology, Stability, and Transfection**

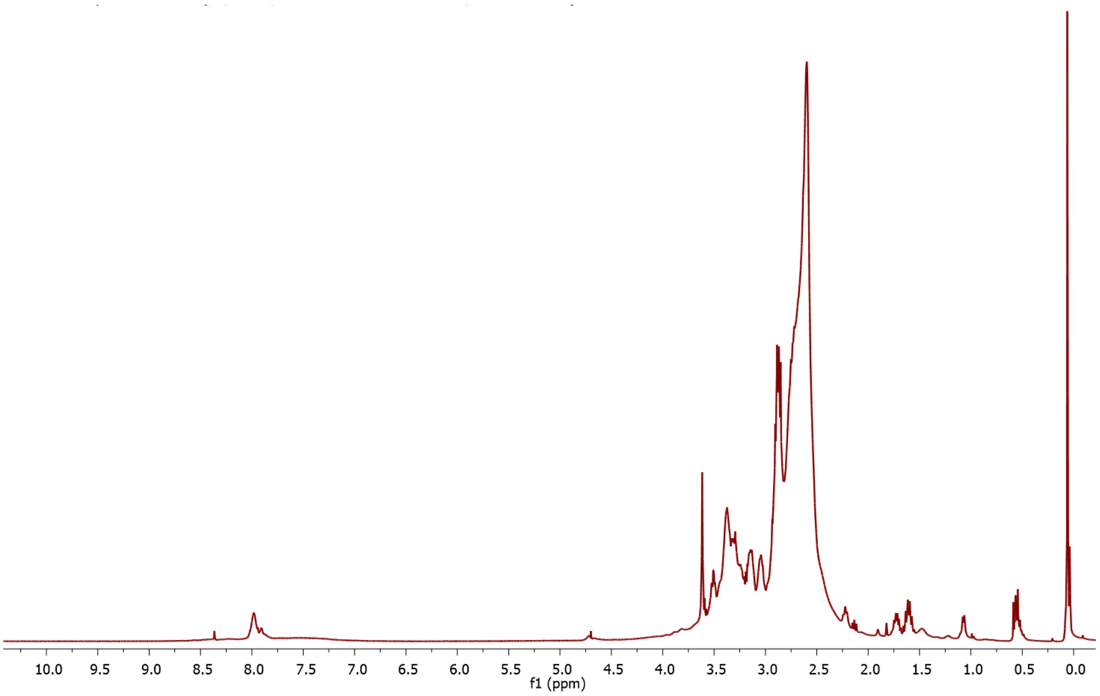


(a)

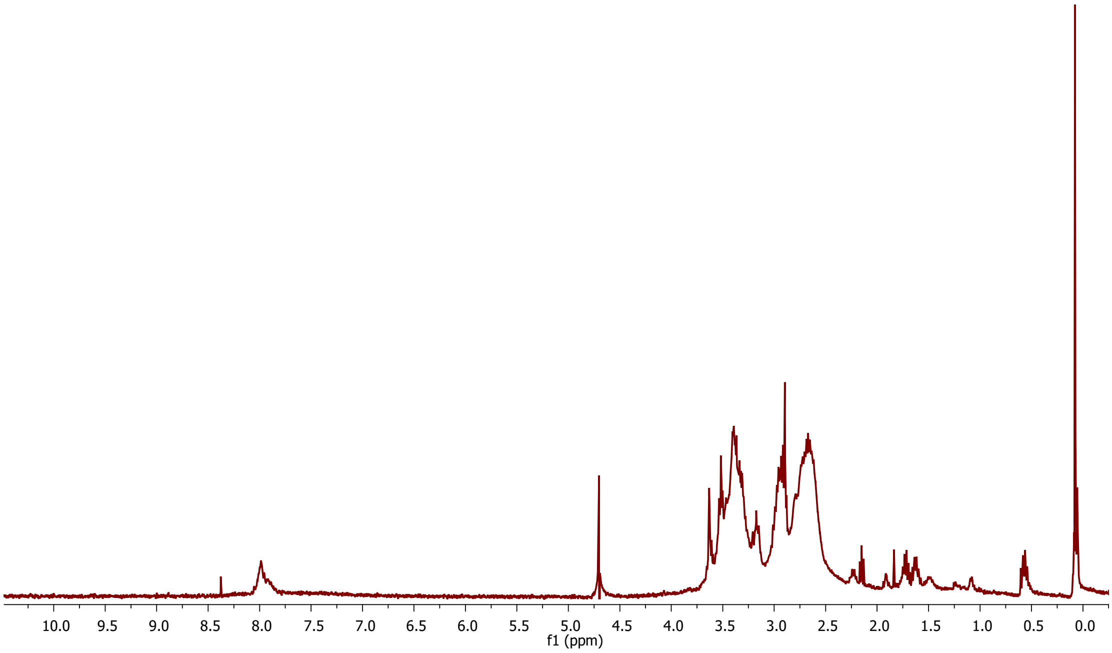


(b)

**Figure S1.** $^{1}$H-NMR spectra of (**a)** TAS-PEI800 in D2O and (**b)** TAS-PEI2000 in D2O

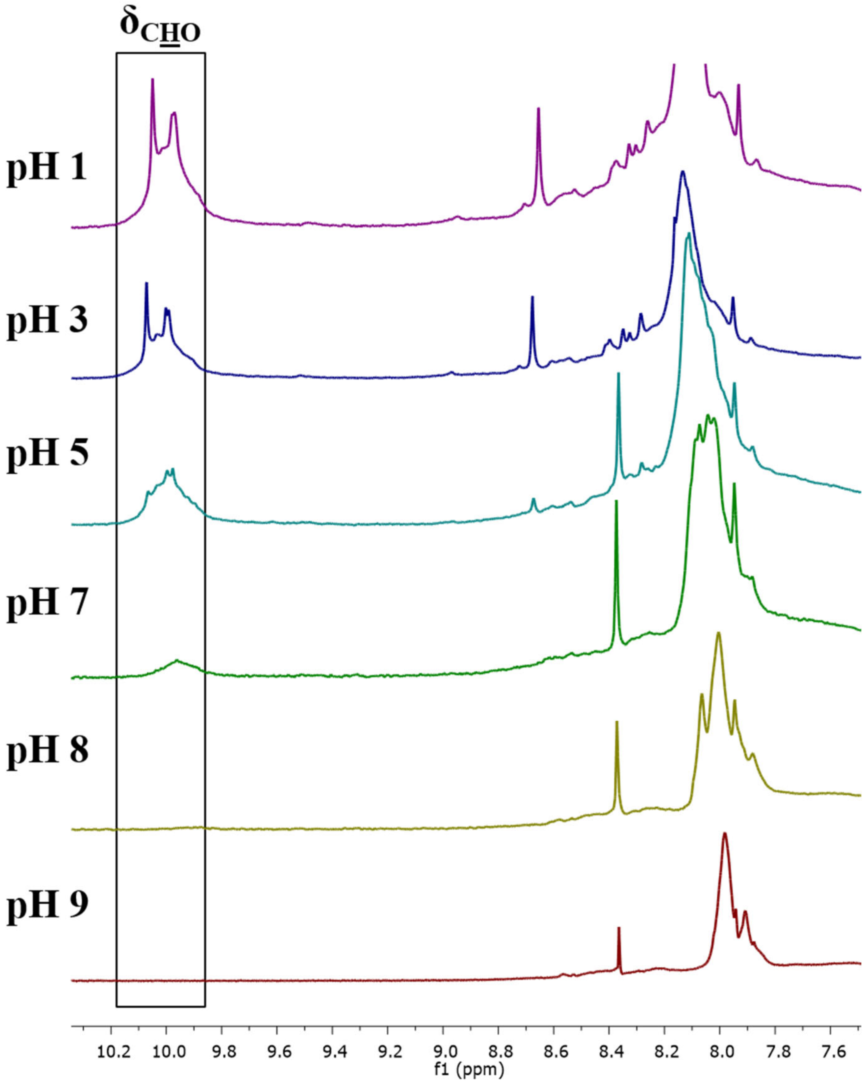


**Figure S2.** $^{1}$H-NMR spectra of TAS-PEI800 in $D_2O$ at different pH values (section)

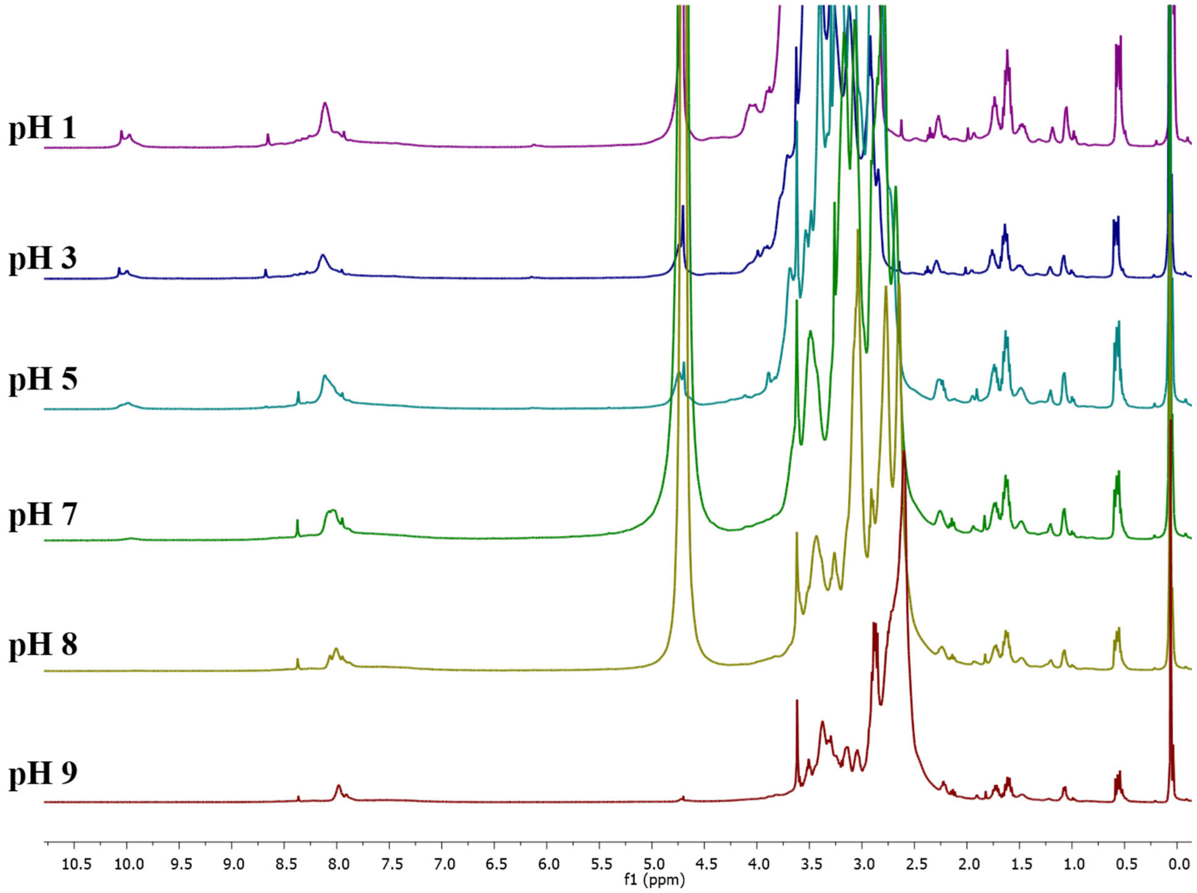


**Figure S3.** $^{1}$H-NMR spectra of TAS-PEI800 in $D_2O$ at different pH values

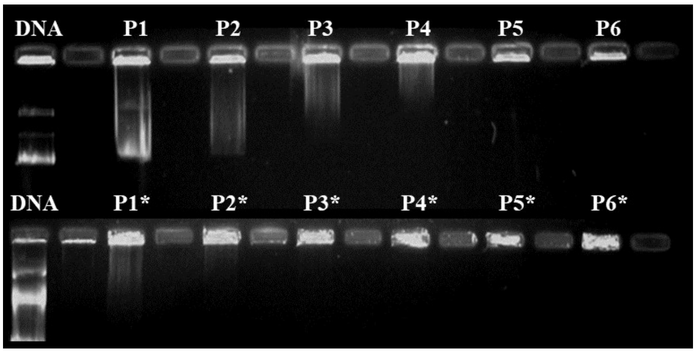


**Figure S4**. Agarose gel electrophoresis for the designed compounds: up - polyplexis generated by **TAS-PEI800** (**P1-P6**) and down - polyplexis generated by **TAS-PEI2000** (**P1*-P6***).
